# Novel Potent Autophagy Inhibitor Ka-003 Inhibits Dengue Virus Replication

**DOI:** 10.3390/v15102012

**Published:** 2023-09-27

**Authors:** Jitra Limthongkul, Kornkamon Akkarasereenon, Tanpitcha Yodweerapong, Poramate Songthammawat, Pirut Tong-Ngam, Alisa Tubsuwan, Nawapol Kunkaew, Phongthon Kanjanasirirat, Tanawadee Khumpanied, Warawuth Wannalo, Sukathida Ubol, Suparerk Borwornpinyo, Poonsakdi Ploypradith, Marisa Ponpuak

**Affiliations:** 1 Department of Microbiology, Faculty of Science, Mahidol University, Bangkok 10400, Thailandsukathida.ubo@mahidol.ac.th (S.U.); 2 Laboratory of Medicinal Chemistry, Chulabhorn Research Institute, Bangkok 10210, Thailandpoonsakdi@cri.or.th (P.P.); 3 Institute of Molecular Biosciences, Mahidol University, Nakhon Pathom 73170, Thailandalisa.tub@mahidol.ac.th (A.T.); 4 Mahidol Vivax Research Unit, Faculty of Tropical Medicine, Mahidol University, Bangkok 10400, Thailand; 5 Excellent Center for Drug Discovery, Faculty of Science, Mahidol University, Bangkok 10400, Thailand; 6 Department of Pathobiology, Faculty of Science, Mahidol University, Bangkok 10400, Thailand; 7 Department of Biotechnology, Faculty of Science, Mahidol University, Bangkok 10400, Thailand

**Keywords:** autophagy, methyl cyclohexene, dengue virus, drug discovery, mulberry

## Abstract

Every year, dengue virus (DENV) affects millions of people. Currently, there are no approved drugs for the treatment of DENV infection. Autophagy is a conserved degradation process that was shown to be induced by DENV infection and required for optimal DENV replication. The modulation of autophagy is, therefore, considered an attractive target to treat DENV infection. This study carried out a high-content image screen analysis using Crispr-Cas9 GFP-LC3 knocked-in HeLa cells of a compound library synthesized from or inspired by natural products and their biocongener precursors to discover novel autophagy inhibitors. The screen identified Ka-003 as the most effective compound for decreasing the number of autophagic vacuoles inside cells upon autophagy induction. Ka-003 could inhibit autophagy in a dose-dependent manner at low micromolar concentrations. More importantly, Ka-003 demonstrated the concentration-dependent inhibition of DENV production in Crispr-Cas9 GFP-LC3 knocked-in THP-1 monocytes. The core structure of Ka-003, which is a methyl cyclohexene derivative, resembles those found in mulberry plants, and could be synthetically prepared in a bioinspired fashion. Taken together, data indicate that Ka-003 hampered autophagy and limited DENV replication. The low cytotoxicity of Ka-003 suggests its therapeutic potential, which warrants further studies for the lead optimization of the compound for dengue treatment.

## 1. Introduction

Dengue virus (DENV), the causative agent of dengue fever (DF) and severe life-threatening dengue hemorrhagic fever/dengue shock syndrome (DHF/DSS), is highly prevalent in tropical and subtropical regions of the world [1]. Global incidences are estimated to account for 100–400 million infections annually, with 21,000 deaths occurring each year [2]. The mechanisms underlying the development of severe dengue remain indeterminate, but are thought to be associated with increased viremia and exacerbated host responses [3]. There are four closely related DENV serotypes (DENV 1–4). Primary infection by any one of the serotypes can provide lifelong immunity and the generation of neutralizing antibodies against that particular serotype [4]. In subsequent infections by the other serotypes, however, cross-reactivity by these antibodies is thought to be one of the prominent risk factors leading to an increased viral titer due to the enhanced infection of the Fcγ receptor (FcγR) expressing cells, such as monocytes [5]. Recently, an additional mechanism responsible for the increased viral titer was reported to be due to DENV-induced autophagy [6,7,8,9,10].

Autophagy is an evolutionary conserved cellular catabolic pathway that delivers cytosolic constituents to lysosomes for degradation. It is used for the clearance of aggregated proteins and damaged cytosolic organelles, as well as to maintain cellular homeostasis [11]. In addition, autophagy also functions as an innate and adaptive immune mechanism against several intracellular bacteria, viruses and parasites [11]. Conversely, autophagy defects have been linked to different diseases, such as cancers, cardiovascular diseases and neurodegenerative disorders [12]. Autophagy can be induced by various conditions, such as cellular starvation [13] and infections [14]. During autophagy, cytoplasmic targets, such as damaged organelles, aggregate proteins and intracellular pathogens, are sequestered into the double-membrane-bound vesicle, called an autophagosome. Then, the outer membrane of the autophagosome fuses with a lysosome to generate an autolysosome, and the sequestered contents are then degraded through lysosomal hydrolases [15,16,17].

In the context of DENV, however, host cell autophagy has been shown to be induced during DENV infection to promote viral production [6,7,8,9,10]. Initially, DENV induces autophagosome formation, but, subsequently, inhibits the fusion of a DENV-associated autophagosome with lysosome, hence, preventing virus degradation [6,9]. The colocalization of the autophagosome marker LC3-II and DENV dsRNA and protein NS1 can be detected in infected cells [10]. In addition, DENV infection was shown to induce autophagy in mice, increasing the viral load in the brain [18]. The mechanisms involved in autophagy-enhanced DENV production include the upregulation of lipophagy, resulting in increased cellular β-oxidation for the generation of ATP required for efficient DENV replication [19], and the increased production of DENV-associated secretory autophagosomes, which carry infectious DENV RNA to recipient cells [20]. Hence, DENV usurps autophagy to increase its viral production. The pharmacological and genetic inhibition of autophagy has been shown to derange infectious DENV production [7,8,19]. As no drug against dengue is yet available, the inhibition of autophagy is seen as a novel drug target for dengue treatment.

As mentioned above, the modulation of autophagy is viewed as an attractive strategy to treat DENV infection. In this work, we set out to identify autophagy inhibitory molecules from compounds synthesized from or inspired by natural products and their biocongener precursors by using a fluorescently based high-content image screening. From the screen, Ka-003 was identified as a compound that effectively decreased the number of autophagic vacuoles in the cells upon autophagy induction. A further characterization of Ka-003 showed that it could inhibit autophagy in a dose-dependent manner at low micromolar concentrations. More importantly, it possessed concentration-dependent anti-DENV activity at low micromolar concentrations. These data indicated the potential development of Ka-003 as a lead compound for the treatment of DENV infection.

## 2. Materials and Methods

### 2.1. Cells, Inhibitors, Antibodies and Fluorescent Dye

HeLa cells (ATCC, Gaithersburg, MD, USA) were maintained in DMEM (Thermo Fisher Scientific Inc., MT, USA), 10% FBS (Thermo Fisher Scientific Inc., Helena, MT, USA) and 4 mM *l*-glutamine (Hyclone Laboratories Inc., Logan, UT, USA) (complete medium) at 37 °C and 5% CO_2_. THP-1 cells (ATCC, MD, USA) were maintained in IMDM (Thermo Fisher Scientific Inc., MT, USA), 10% FBS (Thermo Fisher Scientific Inc., MT, USA), and 4 mM *l*-glutamine (Hyclone Laboratories Inc., UT, USA) (complete medium) at 37 °C and 5% CO_2_. Earle’s balanced salt solution (EBSS; starvation medium) was obtained from Merck, St. Louis, MO, USA. Bafilomycin A1 (Baf; LC laboratories, Woburn, MA, USA) was used at 0.1 µM. For immunoblotting, polyclonal antibodies against P62 (Progen, Heidelberg, Germany) were used at 1:3000 dilutions, polyclonal antibodies against LC3 (MBL International Corporation, Woburn, MA, USA) were used at 1:2000 dilutions, rabbit polyclonal antibodies against DENV NS1 (GeneTex, Irvine, CA, USA) were used at 1:3000 dilutions and monoclonal antibodies against Actin (Abcam, Cambridge, UK) were used at 1:10,000 dilutions. The fluorescent dye Hoechst 33342 (Thermo Fisher Scientific Inc., MT, USA) was used at 1:500 and a monoclonal antibody against DENV E (clone 4G2; ATCC, MD, USA) was used at 1:100 dilutions.

### 2.2. Generation and Validation of the CRISPR-Cas9 GFP-LC3 KI HeLa and THP-1 Cells

GFP-LC3 KI HeLa cells were generated using the CRISPR-Cas9 system in combination with a donor plasmid template containing the eGFP gene flanked by a 489 bp left homology arm (LHA) and a 573 bp right homology arm (RHA) of the LC3 gene. The specific single-guide RNA (sgRNA) that targets the start codon of the LC3 gene (5′-AGATCCCTGCACCATGCCGT-3′) was designed using http://crispor.tefor.net/accessed on 8 September 2022 and cloned into the Bbs1 site of the pSpCas9 (BB)-2A-Puro V2.0 (Addgene, Watertown, MA, USA; plasmid ID 48139) following the protocol described previously [21]. HeLa or THP-1 cells were cotransfected with 2.5 µg of plasmid-expressing Cas9 and the sgRNA and 2.5 µg of the donor template using the Amaxa Nucleofector IIb. One week after transfection, GFP-positive cells were sorted into a 5 mL polystyrene round-bottom tube (Corning Inc., Corning, NY, USA) using a BD FACSAria III (Becton Dickinson, Hector Lake, NJ, USA). Single-cell clones were isolated with a limited dilution in a 96-well plate and for subsequent expansion for gene knocked-in validation. To confirm the insertion of the eGFP gene into the LC3 locus, total RNAs were extracted from each GFP-positive cell clone and converted into cDNAs using the RevertAid First Strand cDNA Synthesis Kit (Thermo Fisher Scientific Inc., MT, USA), according to the manufacturer’s instructions. The PCR amplification of a full-length eGFP-LC3 fragment consisting of 1768 nucleotides was carried out using Phusion^®^ High-Fidelity DNA Polymerase (New England BioLabs, Ipswich, MA, USA) and a primer pair (F1: 5′-TGCGGGCTGAGGAGATACAA-3′ and R1: 5′-CCGTTTACCCTGCGTTTGTG-3′). The PCR product was purified and, subsequently, cloned into pGEM-T Easy (Promega, Fitchburg, WI, USA). The full-length eGFP-LC3 fragment was confirmed with Sanger sequencing.

### 2.3. Screening of Compounds

The screening of natural-product-derived compounds and their derivatives for autophagy inhibitory activity was conducted by using the Operetta high-content imaging analysis system (PerkinElmer Corporation, Waltham, MA, USA) to quantitate the number of GFP-LC3^+^ puncta (a cellular marker for autophagic vacuoles) in GFP-LC3 KI HeLa cells. In brief, GFP-LC3 KI HeLa cells (6 × 10^3^ cells/well) were plated into each well of 96-well plates overnight. Starvation was performed by washing the corresponding wells three times with PBS. The cells were then treated with DMSO, starvation (EBSS media; autophagy induction control) + DMSO, or starvation (EBSS media) + 50 µM final concentration of each compound for 2 h. A total of 57 compounds were screened in this study. They were the intermediates that were synthesized for the total synthesis of palodesangrens [22]. The cells were then fixed with 4% paraformaldehyde for 10 min and stained with Hoechst for 15 min. The cells were then analyzed through a high-content image analysis to quantify the number of fluorescent GFP-LC3^+^ autophagic puncta per cell, as previously described [23]. Compounds that could down-regulate the number of autophagic puncta to be less than the mean of the DMSO control were identified as positive compounds for autophagy inhibitors.

### 2.4. Ka-003 Synthesis and Structure Characterization

Ka-003 synthesis was conducted as previously described [22]. In brief, a solution of arylidene (457.3 mg, 1.33 mmol, 1.0 equiv) and chalcone (439.5 mg, 1.06 mmol, 0.8 equiv) in toluene (2.6 mL) was placed in a sealed tube under magnetic stirring at 110 °C using an oil bath for 48 h. The cooled reaction mixture was then concentrated under reduced pressure and the crude product was chromatographically purified over silica gel (30−40% EtOAc/hexane) to produce the desired cycloadduct as a yellow foamy solid (393.0 mg, 0.52 mmol, 65%) of a 1:0.9 (*endo*:*exo*) mixture of isomers. Further purification using column chromatography on silica then furnished the desired *endo*isomer. ^1^H NMR (300 MHz, CDCl_3_) δ 7.75 (dd, *J* = 8.5, 2.0 Hz, 1H), 7.69 (d, *J* = 2.0 Hz, 1H), 7.42 (s, 1H), 7.25 (d, *J* = 8.4 Hz, 1H), 6.99 (d, *J* = 8.2 Hz, 1H), 6.83 (s, 1H), 6.54–6.49 (m, 1H), 6.46 (d, *J* = 2.2 Hz, 1H), 5.48 (brs, 1H), 5.21 (d, *J* = 6.6 Hz, 1H), 5.15 (d, *J* = 6.6 Hz, 1H), 4.63 (d, *J* = 7.0 Hz, 1H), 4.61–4.54 (m, 1H), 4.30 (brs, 1H), 4.17 (d, *J* = 6.9 Hz, 1H), 3.60 (s, 3H), 3.51 (s, 3H), 3.07 (s, 3H), 2.38–2.33 (m, 2H), 2.32 (s, 6H), 2.25 (s, 3H), 1.88 (s, 3H). ^13^C NMR (75 MHz, CDCl_3_) δ 198.0, 169.4, 168.1, 167.8, 157.6, 155.4, 153.3, 149.7, 145.2, 142.1, 137.3, 136.1, 134.5, 125.9, 124.4, 123.2, 122.9, 122.1, 113.2, 104.8, 104.0, 102.72, 95.40, 94.7, 56.5, 55.9, 55.3, 49.3, 37.1, 37.0, 23.3, 21.5, 20.7, 20.6. ESI-HRMS: calcd for C_37_H_39_O_12_^79^BrNa (M + Na^+^), 777.1517; found, 777.1514; calcd for C_37_H_39_O_12_^81^BrNa (M + Na^+^), 779.1502; found 779.1495.

### 2.5. SDS-PAGE and Immunoblotting

The cells were lysed in a lysis buffer containing 62.5 mM Tris-HCl (pH 6.8), 10% glycerol, 2% SDS, 5% β-mercaptoethanol and 0.01% bromophenol blue. The cell lysates were then separated with 15% polyacrylamide gels and transferred onto nitrocellulose membranes (Amersham Biosciences, Amersham, UK). The membranes were then blocked with a 5% blocking solution (Roche Diagnostics, Indianapolis, IN, USA) for 1 h at room temperature, followed by incubation with primary antibodies against LC3, P62, DENV NS1 or Actin at 4 °C overnight. The membranes were washed 4 times with 0.1% PBST and incubated with appropriate horseradish peroxidase-conjugated secondary antibodies for 1 h at room temperature. The membranes were then washed 4 times; the expression levels of the proteins were detected using the chemiluminescence method (Roche Diagnostics). The Western blot image was then exported to ImageJ 1.54d software and the band density was analyzed. Each protein band density was then normalized to that of Actin and used as the loading control.

### 2.6. Plaque Assays

Plaque assays were performed in the presence of varying compound concentrations to examine the effect of the compounds on the virus life cycle, which included viral replication and assembly into infectious particles. Briefly, GFP-LC3 KI THP-1 cells (5 × 10^5^) were plated onto each well of 12-well cell culture plates. After 16 h, the cells were infected with the DENV 2 strain 16681 in MEM medium supplemented with 2% FBS (MOI of 1). After 1.5 h, the virus inoculum was aspirated and washed with PBS three times, after which IMDM with 5% FBS was added with various drug concentrations (0.78–25 μM). The control cells were treated with 1% DMSO in the absence of any drug. After an additional 48 h, the supernatants were collected and analyzed with a plaque assay. In brief, Vero cells were plated (2.5 × 10^4^/100 uL/well) in 96-well plates and incubated overnight. Serially diluted supernatants from DENV-2-infected GFP-LC3 KI THP-1 cells treated with Ka-003 at different concentrations, as described above, were incubated with cells in duplicate wells for 1.5 h. The cells were overlaid with 1× MEM medium supplemented with 5% FBS containing 1.2% microcrystalline cellulose (Avicel^®^; Merck, St. Louis, MO, USA) and were incubated for 3 d at 37 °C. The plaques were visualized by fixing the cell monolayers and staining them with crystal violet solution [24]. The plaques were counted and the values of plaque-forming units (PFU) per mL were determined.

### 2.7. Dengue RNA Quantitation Using Real-Time PCR

GFP-LC3 KI THP-1 cells were mock-infected or infected with the DENV 2 strain 16681 at MOI of 1 for 1.5 h, followed by treatment with compounds. At 24 h postinfection, the supernatants were collected and the DENV 2 genome copy numbers were quantified with qRT-PCR using the KAPA SYBR FAST qPCR Kit 2X Master MIX (Kapa Biosystems Inc., Wilmington, MA, USA) Total RNAs were extracted using the Trizol reagent (Thermo Fisher Scientific), after which the cDNAs were synthesized using random hexamer primers and AMV reverse transcriptase (Promega, Fitchburg, WI, USA). The amplification was performed using specific primers to the DENV polyprotein gene, namely, DV2-F: 5′-CAATATGCTGAAACGCGAGAG-3′ and DV2-R: 5′-GTGGGATTGTTAGGAAACGAA-3′. The DENV 2 copy number was calculated from the cycle threshold value of the amplification plot. The viral RNA genome expression was then normalized against GAPDH- and DMSO-treated control cells, respectively, according to the following equation: ∆∆Ct = ∆Ct (DV2 target gene) − ∆Ct (DMSO).

### 2.8. Immunofluorescence Assay

The GFP-LC3 KI THP-1 cells were plated into each well of 96-well black plates at a density of 5 × 10^4^ cells/well overnight. The cells were then infected with the DENV 2 strain 16681 at MOI of 5 for 1.5 h. The cells were washed and subjected to the indicated treatment for 24 h. The cells were fixed with ice-cold methanol/acetone and probed with the anti-DENV E protein antibody (clone 4G2) at a dilution of 1:100, followed by the secondary antibody labelled with Alexa-568 at a dilution of 1:400, together with a 1:500 dilution of Hoechst. The number of GFP-LC3^+^ puncta per cell and DENV E protein fluorescence intensity per cell were then analyzed with high-content imaging, as described previously [25], except for channel Alexa-568 being used to calculate the intensity properties while channel Alexa-488 was used to find spots.

### 2.9. Statistical Analysis

Unless otherwise stated, all the experiments were conducted at least three times and the data were pooled for the determination of the mean± standard error of the mean (S.E.M.). All data were analyzed using Prism software (GraphPad 9.0) using one-way ANOVA with Tukey’s multiple comparison testing. Statistical significance was considered to be indicated at *p*-values less than 0.05.

## 3. Results

### 3.1. Identification of Ka-003 as an Autophagy Inhibitory Compound

**Figure 1:**
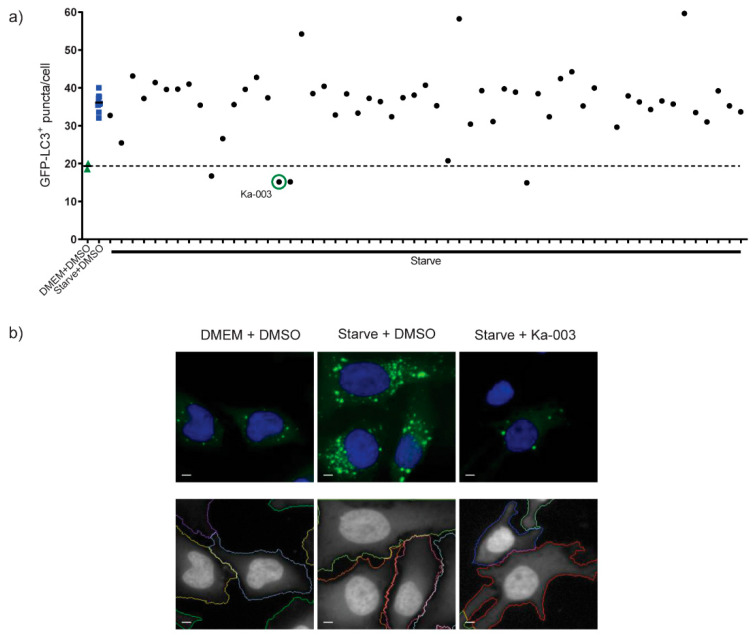
**Screening of compounds for autophagy inhibitory activity** (**a**,**b**). GFP-LC3 knocked-in HeLa cells (6 × 10^3^ cell per well) were plated into each well of 96-well black plates overnight. Cells were treated for 2 h with DMEM + DMSO (negative control), starvation (EBSS) + DMSO (autophagy induction control) or 50 μM of each compound in starvation media (EBSS). Cells were then fixed with 4% paraformaldehyde and the nuclei were stained with Hoechst 33342. Cells were then analyzed using high-content image analysis to quantify the number of total GFP-LC3^+^ puncta per cell. The dashed line represents the mean of DMEM + DMSO control. Ka-003 was identified as the most effective compound to decrease the number of GFP-LC3^+^ puncta per cell. Representative images of the high-content image analysis with the boundary of cells (below panels) are shown (Bar 5 μm).

Since autophagy has been shown to be induced during DENV infection and required for optimal DENV replication [6,7,8,9,10], and since Thai herbal and natural-product-based traditional medicines have been used to treat diseases, we determined whether autophagy inhibitory activity could be found in these molecules and their derivatives. To search for such compounds, Crispr-Cas9 GFP-LC3 knocked-in HeLa cells (GFP-LC3 KI HeLa) (Figure S1) were initially generated to be used in the high-content image screening assay. It should be noted that LC3 was used as a biological marker for autophagic vesicles inside cells [26]. LC3 was cleaved at the C-terminus soon after the translation to generate cytoplasmic LC3-I, which became LC3-II, decorating the inner and outer membranes of autophagic vesicles upon being lipidated with phosphatidylethanolamine [27]. We then tested whether the autophagy process inside the GFP-LC3 KI HeLa cells proceeded normally by treating the cells with starvation (autophagy induction control) in the presence or absence of the standard autophagy inhibitor wortmannin, which inhibits autophagosome formation, or autophagic flux inhibitor bafilomycin A1, which inhibits autophagosome–lysosome fusion. Wild-type (WT) HeLa cells were used as the control. An increase in GFP-LC3^+^ puncta was observed upon the starvation of the GFP-LC3 KI HeLa cells (Figure S1). Furthermore, the addition of wortmannin decreased the GFP-LC3^+^ puncta in the starved GFP-LC3 KI HeLa cells, while bafilomycin A1 increased the GFP-LC3^+^ puncta (Figure S1). Similar autophagy responses upon the treatment of the WT HeLa cells with starvation, starvation + wortmannin and starvation + bafilomycin A1 were observed, albeit with lower levels when compared to those seen in the GFP-LC3 KI HeLa cells. These data indicated that the autophagy responses in the GFP-LC3 KI HeLa cells appeared to be normal. Subsequently, GFP-LC3 KI HeLa cells were used in the high-content image analysis screen for this study. In brief, the GFP-LC3 KI HeLa cells were treated with DMEM + DMSO (negative control), starvation + DMSO (autophagy induction control) or starvation in the presence of 50 µM of each compound for 2 h. The cells were then processed for the high-content image analysis. The number of the total GFP-LC3^+^ puncta per cell was then quantified. Ka-003 was identified as the most effective compound for reducing the number of GFP-LC3^+^ puncta per cell from the screen (Figure 1a,b).

### 3.2. Ka-003 Effectively Inhibits Autophagosome Formation in a Dose-Dependent Manner

**Figure 2:**
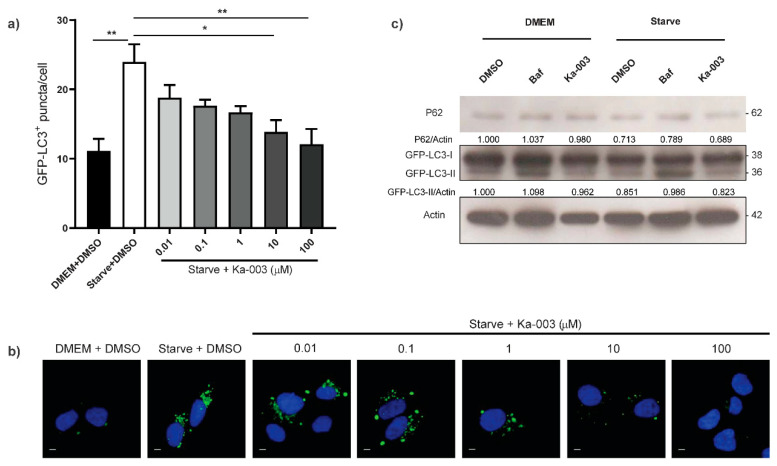
**Ka-003 potently inhibited autophagy**. (**a**,**b**) GFP-LC3 KI HeLa cells were plated onto 96-well black plates and then untreated or treated with DMSO, starvation + DMSO or starvation + Ka-003 at the indicated concentrations for 2 h. Cells were then processed for high-content image analysis. The number of GFP-LC3^+^ puncta per cell was then analyzed. Data are the means ± SEM from three independent experiments; * *p* < 0.05 and ** *p* < 0.01, all relative to the starvation + DMSO control were determined using one-way ANOVA with Tukey’s multiple comparison test (Ka-003 IC_50_ = 5.05 μM). Bar 5 μm. (**c**) Ka-003 reduced GFP-LC3-II protein levels in GFP-LC3 KI HeLa cells. The cells were treated with DMSO, 1 μM bafilomycin A1 (Baf) or 6.25 μM of Ka-003, both in DMEM and starvation media for 2 h. Cells were then harvested for immunoblot analysis of P62 and GFP-LC3 proteins. Representative images cropped from the same blot are shown.

To confirm the autophagy inhibitory activity of Ka-003 and determine the effective concentrations at which it could hamper autophagy, we performed a more thorough analysis of the GFP-LC3^+^ puncta per cell in the GFP-LC3 KI HeLa cells upon treatment with different concentrations of Ka-003 under starvation conditions. The results showed that Ka-003 decreased the number of GFP-LC3^+^ puncta per cell when compared to that of the DMSO-treated starved control cells in a dose-dependent manner, indicating that it acted by inhibiting autophagosome formation (Figure 2a,b). Ka-003 dampened the number of GFP-LC3^+^ puncta per cell, starting at a concentration as low as 10 nM under starvation conditions. At 100 μM under starvation conditions, Ka-003 could reduce the number of autophagic vacuoles to similar levels to those seen in the DMSO-treated nonstarved cells (Figure 2a,b). The IC_50_ concentration of Ka-003 in autophagy inhibition was calculated to be 5.05 μM. In addition, P62 and LC3-II protein levels were examined through immunoblotting to confirm that Ka-003 inhibited autophagy. Unlike LC3-II, which decorated both the inner and outer membranes of autophagosomes, and the inner membrane population of LC3-II was degraded in the autolysosome, while the outer membrane population of LC3-II was recycled back to the cytosol [28,29], P62 was engulfed into the autophagosome and was a true substrate subjected to autophagy-mediated degradation [30]. The levels of the aforementioned proteins in the immunoblot analysis were used to evaluate the autophagic activity inside the cells [26]. To determine this, the GFP-LC3 KI HeLa cells were treated with DMSO (negative control), bafilomycin A1 (autophagic flux inhibitor control) or 6.25 μM of Ka-003 in both DMEM and starvation media for 2 h. As expected, we detected increased P62/Actin and GFP-LC3-II/Actin levels upon the treatment of the GFP-LC3 KI HeLa cells with bafilomycin A1 when compared to those of cells treated with DMSO, both in DMEM and starvation media conditions (Figure 2c). In addition, GFP-LC3-II/Actin levels in the Ka-003-treated cells when compared to those of cells treated with DMSO, both in DMEM and starvation media conditions, appeared to decrease (Figure 2c). Furthermore, we did not detect an increase in P62/Actin levels upon the treatment of the cells with Ka-003 when compared to those of the DMSO-treated control cells, which indicated that Ka-003 was not an autophagic flux inhibitor. These results confirmed that Ka-003 acted by inhibiting autophagosome formation. Thus, Ka-003 was shown to be a novel potent autophagy inhibitor.

### 3.3. Ka-003 Restricts DENV Production in a Dose-Dependent Manner in THP-1 Monocytic Cells

**Figure 3:**
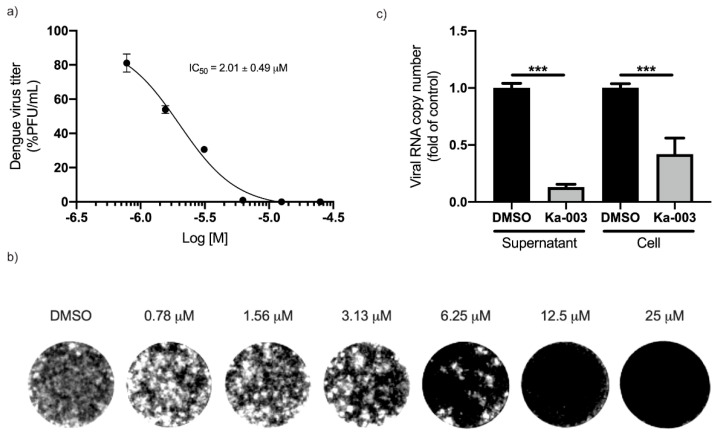
**Ka-003 exhibited dose-dependent anti-DENV activity** (**a**,**b**). GFP-LC3 KI THP-1 cells were infected with DENV 2 at MOI of 1 for 1.5 h. Cells were washed and then treated with varying concentrations of Ka-003. At 48 h after incubation, the supernatants were collected. The amounts of infectious virions were then determined with plaque assay using Vero cells. Data are the means ± SEM from three independent experiments. The IC_50_ value of Ka-003 was then determined using GraphPad Prism software (Ka-003 IC_50_ = 2.01 ± 0.49 μM). (**c**) GFP-LC3 KI THP-1 cells were infected with DENV 2 at MOI of 1 for 1.5 h. Cells were washed and then treated with 6.25 μM of Ka-003 or DMSO for 24 h. Supernatants were collected and infected cells were then harvested. The number of viral RNAs in the infected cells and supernatants were then analyzed with qRT-PCR. Data are the means ± SEM from three independent experiments; *** *p* < 0.001, all relative to the DMSO control, were determined using one-way ANOVA with Tukey’s multiple comparison testing.

As Ka-003 can potently inhibit autophagy, and autophagy is required for optimal DENV replication, we tested whether Ka-003 could dampen DENV production in THP-1 monocytic cells, used as a host cell model for DENV infection. To determine this, we generated CRISPR-Cas9 GFP-LC3 knocked-in THP-1 (GFP-LC3 KI THP-1) cells (Figure S2). The autophagy responses in the GFP-LC3 KI THP-1 cells appeared to be unaffected and exhibited normal function similar to those observed in the WT THP-1 cells used as the control (Figure S2). Upon the starvation of the GFP-LC3 KI THP-1 cells, an increase in the GFP-LC3^+^ puncta was observed, while the addition of wortmannin reduced the GFP-LC3^+^ puncta in starved GFP-LC3 KI THP-1 cells and bafilomycin A1 augmented the GFP-LC3^+^ puncta, respectively (Figure S2). The GFP-LC3 KI THP-1 cells were then infected with DENV 2 at MOI of 1 for 1.5 h. The cells were washed to eliminate the extracellular virus, followed by treatment with two-fold serial dilutions of Ka-003. At 48 h after incubation, the supernatants were collected. The amounts of infectious virions produced were then determined through a plaque assay. The results showed that Ka-003 could effectively inhibit DENV production in GFP-LC3 KI THP-1 cells in a concentration-dependent manner with low micromolar concentrations (Ka-003 IC_50_ = 2.01 ± 0.49 μM; the plaque number at IC_50_ concentration = 51,030 ± 1483 PFU/mL) (Figure 3a,b). From this graph, 6.25 μM of Ka-003 was selected and used in subsequent experiments, as, at this concentration, an inhibition of more than 90% of the infectious virion production was observed. We then assessed the DENV genome production through the use of qRT-PCR, both in host cells and supernatants, after treatment with 6.25 μM of Ka-003. The results showed that Ka-003 decreased the DENV RNA copy numbers inside the host cells and in the supernatants (viral RNA copy numbers in the supernatant: DMSO = 1,006,000 ± 29,143 copies/μL and Ka-003 = 89,633 ± 4317 copies/μL; viral RNA copy numbers in the cell: DMSO = 64,467 ± 4253 copies/μL and Ka-003 = 28,533 ± 2397 copies/μL) (Figure 3c), confirming that Ka-003 effectively restricted DENV. Furthermore, Ka-003 possesses low cytotoxicity towards normal human kidney HK-2 cells (CC_50_ = 32.05 ± 1.58 μM), GFP-LC3 KI HeLa cells (CC_50_ = 98.27 ± 5.34 μM) and GFP-LC3 KI THP-1 cells (CC_50_ = 42.78 ± 3.11 μM) (Figures S3 and S4). These results suggested a therapeutic potential, warranting further studies on the lead optimization of Ka-003 for dengue treatment.

### 3.4. Causal Relationship between Autophagy Inhibition Using Ka-003 and DENV Restriction

**Figure 4:**
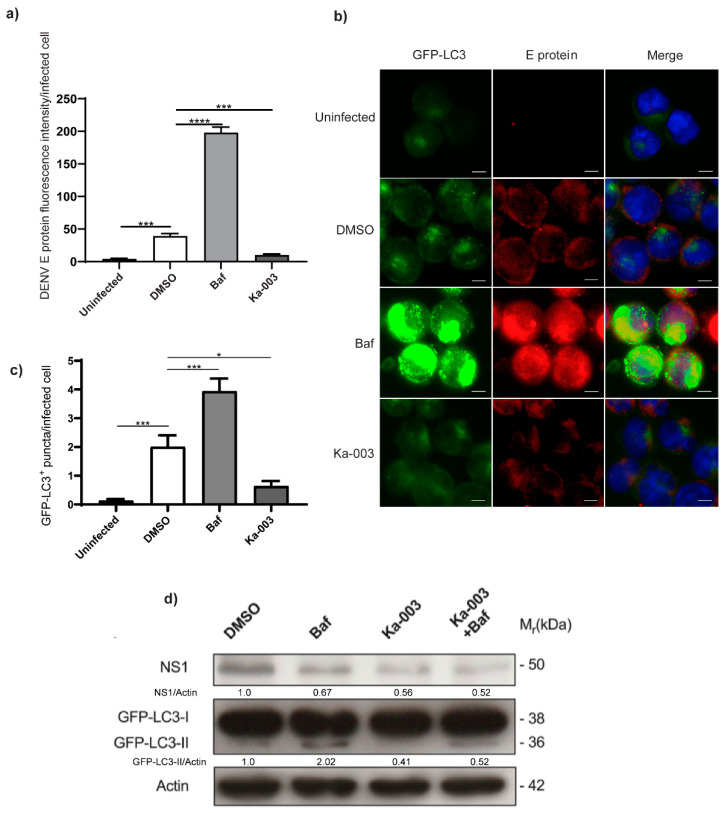
**Autophagy inhibition using Ka-003 in correlation with its anti-DENV activity** (**a**–**c**). Ka-003 decreased GFP-LC3^+^ puncta and DENV E protein fluorescence intensity in the infected cells. GFP-LC3 KI THP-1 cells were infected with DENV 2 at MOI of 5 for 1.5 h. Cells were then treated with DMSO, 0.1 μM of Baf or 6.25 μM of Ka-003 for 24 h. Cells were then fixed and stained with anti-DENV E protein antibody clone 4G2 (ATCC:HB-112). The samples were then processed for high-content image analysis. Data are means ± SEM from three independent experiments; * *p* < 0.05, *** *p* < 0.001 and **** *p* < 0.0001, all relative to the DMSO control, determined using one-way ANOVA with Tukey’s multiple comparison testing. (**d**) Ka-003 reduced the amounts of GFP-LC3-II and DENV NS1 proteins in the infected cells. GFP-LC3 KI THP-1 cells were infected with DENV 2 at MOI of 5 for 1.5 h. Cells were washed and then treated with DMSO, 0.1 μM of Baf or 6.25 μM of Ka-003, with or without Baf, for 4 h. Cells were then harvested for immunoblot analysis of the DENV NS1 and host GFP-LC3 proteins. Representative images cropped from the same blot are shown.

To investigate the causal relationship between autophagy inhibition using Ka-003 and DENV restriction, we determined whether autophagy inhibition using Ka-003 was correlated with DENV inhibition in GFP-LC3 KI THP-1 cells. To determine this, the GFP-LC3 KI THP-1 cells were infected with DENV 2 and then treated with DMSO, bafilomycin A1 or Ka-003 for 24 h. The cells were then fixed and stained with an anti-DENV E protein antibody. The number of GFP-LC3 and level of DENV E protein fluorescence intensity per infected cell in different conditions were then quantified and compared with the high-content image analysis. An increase in the number of GFP-LC3^+^ puncta was detected in DENV-infected DMSO-treated cells (Figure 4a,c), consistent with previous findings that DENV infection increases autophagy in the host cells [6,7,8,9,10]. As expected, a further increase in the GFP-LC3^+^ puncta was observed after the treatment of the cells with bafilomycin A1 (Figure 4a,c). In agreement with the findings in the current study, the treatment of DENV-infected GFP-LC3 KI THP-1 cells with Ka-003 reduced the number of GFP-LC3^+^ puncta in the host cells (Figure 4a,c), which correlated with the decreased level of DENV E protein fluorescence intensity in these cells (Figure 4a,b). We also confirmed the aforementioned results by performing a WB analysis of the DENV NS1 protein in DENV-infected GFP-LC3 KI THP-1 cells. Decreased levels of GFP-LC3-II and DENV NS1 proteins were observed in DENV-infected Ka-003-treated cells when compared to those observed in the DMSO-treated control cells (Figure 4d). Thus, a correlation was shown in the data between autophagy inhibitory and anti-DENV activities using Ka-003. Of note, while the bafilomycin A1 treatment increased the level of DENV E protein fluorescence intensity in the infected cells (Figure 4a)—in agreement with previous studies showing that DENV induced autophagosome formation, but inhibited DENV-associated autophagosomes to fuse with the lysosomes and, hence, spared the virus from lysosomal degradation [9]—we observed a decrease in NS1 protein upon the treatment of the infected cells with bafilomycin A1. The reason for the decreased NS1 protein level in the bafilomycin-A1-treated cells was unclear. Perhaps bafilomycin A1 affected the translation of the NS1 protein through an unknown mechanism.

### 3.5. Ka-003 Is a Derivative of Methyl Cyclohexene and the Endoisomer Exhibits Autophagy Inhibitory and Anti-DENV Activities

**Figure 5:**
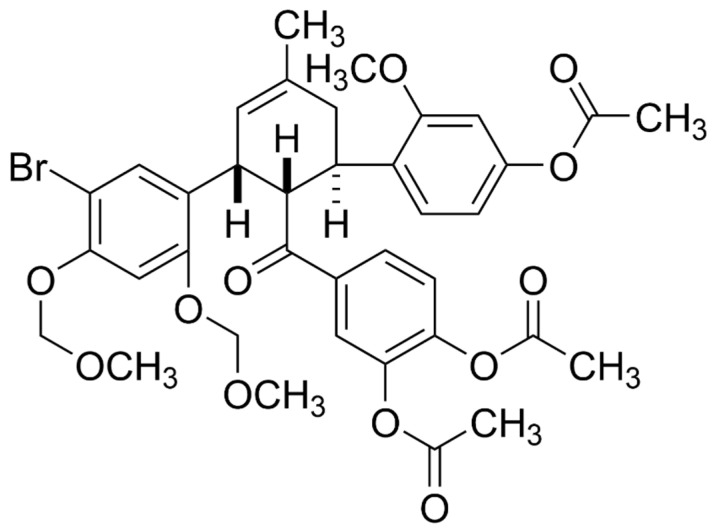
**Chemical structure of Ka-003.** The newly identified potent natural-product-derived autophagy inhibitor, Ka-003, is a derivative of methyl cyclohexene with the core structurally related to several natural products from mulberry. The Diels–Alder reaction between the corresponding arylidene and chalcone furnished 4-(4″-acetoxy-5-bromo-2″-methoxy-2,4-bis(methoxymethoxy)-5′-methyl-1′,2′,3′,4′tetrahydro-[1,1′:3′,1″-terphenyl]-2′-carbonyl)-1,2-phenylene diacetate (Ka-003).

**Figure 6:**
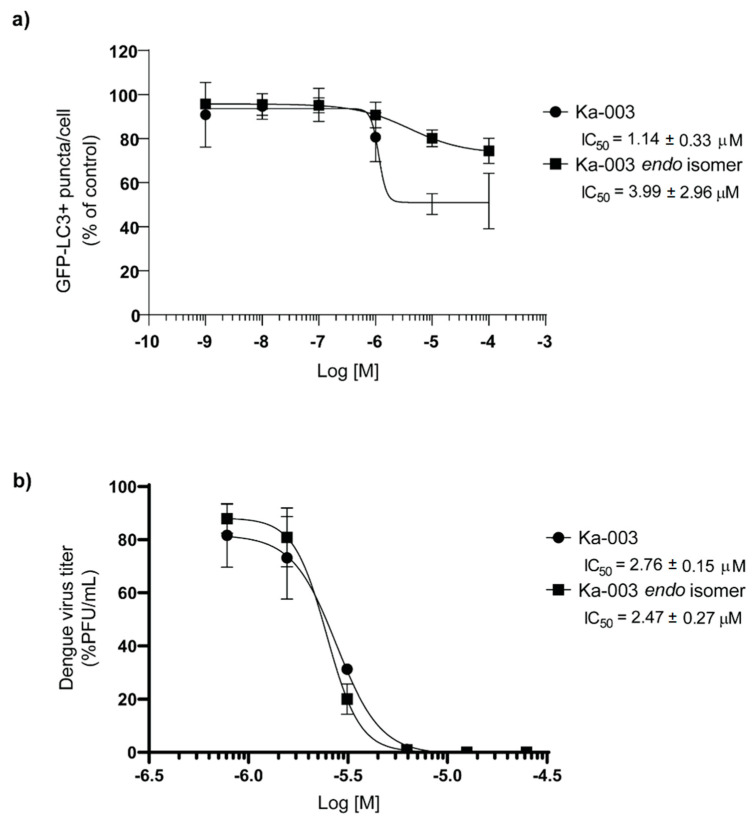
**Ka-003 *endo* isomer possesses autophagy inhibitory and anti-DENV activities similar to those of the original Ka-003**. (**a**) GFP-LC3 KI HeLa cells were plated onto 96-well black plates and then untreated or treated with DMSO or varying concentrations of Ka-003 or Ka-003 *endo* isomer for 2 h. Cells were then processed for high-content image analysis. The number of GFP-LC3^+^ puncta per cell was then analyzed. Data were the means ± SEM from three independent experiments. IC_50_ values were then determined using GraphPad Prism software. (**b**) GFP-LC3 KI THP-1 cells were infected with DENV 2 at MOI of 1 for 1.5 h. Cells were washed and then treated with DMSO or varying concentrations of Ka-003 or Ka-003 *endo* isomer. At 48 h after incubation, the supernatants were collected. The amounts of infectious virions were then determined through a plaque assay using Vero cells. Data are the means ± SEM from three independent experiments. IC_50_ values were then determined using GraphPad Prism software.

The structure of Ka-003 was determined through the use of spectroscopic methods (^1^H-NMR, ^13^C-NMR and MS analysis) (please see the Materials and Section 2 for more information). The core structure of Ka-003 is a derivative of methyl cyclohexene, most directly accessible via the Diels–Alder reaction of the corresponding arylidene and chalcone. The detailed preparation followed the developed synthetic method reported previously [22]. The compound was obtained as a *circa* 1:1 mixture of the *endo-* and *exo*isomers with a 65% overall yield (Figure 5). Since there were two isomers present, we further determined which isomer was responsible for the observed autophagy inhibitory and anti-DENV activities. The further purification of the two isomers through column chromatography on silica furnished only the *endo*isomer in its pure form, while the *exo*isomer remained contaminated with the *endo*isomer. We then compared the autophagy inhibitory and anti-DENV activities between the original Ka-003 containing both the *endo-* and *exo*isomers with that of the purified Ka-003 *endo*isomer (Figure 6a,b). The results showed that the IC_50_ value for the autophagy inhibitory activity of the original Ka-003 was comparable to that of the Ka-003 *endo*isomer (IC_50_ = 1.14 ± 0.33 μM and IC_50_ = 3.99 ± 2.96 μM, respectively). Furthermore, the anti-DENV activity of the original Ka-003 was similar to that of the Ka-003 *endo*isomer (IC_50_ = 2.76 ± 0.15 μM and IC_50_ = 2.47 ± 0.27 μM, respectively). Therefore, the Ka-003 *endo*isomer appeared to be responsible for the autophagy inhibitory and anti-DENV activities.

In summary, the current study showed that Ka-003 is a novel effective autophagy inhibitor that can restrict DENV production at low micromolar concentrations. It also possesses low cytotoxicity towards normal human cells. The ability of Ka-003 to potently decrease DENV production indicates the significant potential of this compound to be developed further as a lead molecule for the treatment of DENV infection.

## 4. Discussion

Due to the requirement of autophagy for optimal DENV replication [6,7,8,9,10], the search for small molecules that possess autophagy inhibitory activity to be used for dengue treatment is of high interest. As many herbal and natural-product-derived compounds have been used in traditional medicines to treat diseases, they hold notable pharmacological potential, though the underlying mechanisms possessed by these compounds remain largely uncharacterized. This study aimed to identify the natural-product-derived compounds and their derivatives exhibiting autophagy inhibitory activity by using phenotypic-based screening. The high-content image analysis screen identified Ka-003, a derivative of methyl cyclohexene (Figure 5), resembling those found in mulberry plants [31,32,33,34,35], as enabling a potent decrease in the number of autophagic vacuoles in cells in a dose-dependent manner (Figure 1 and Figure 2). More importantly, upon the application of the Ka-003 treatment to DENV-infected cells, a dose-dependent decrease in DENV infectious particle production was observed (Figure 3 and Figure 6). The Ka-003 treatment also reduced the levels of DENV RNA in the infected cells and supernatants, and decreased the levels of DENV E and NS1 proteins in the host cells (Figure 3 and Figure 4). Ka-003 also showed low toxicity towards normal human cells and cells used in this study (Figures S3 and S4). Currently, there is no approved drug available to treat DENV infection, and very few compounds have been tested in clinical trials, with no positive effect having, thus far, been reported. Thus, further studies on Ka-003 are warranted for further development as a lead compound for dengue treatment.

As mentioned above, host cell autophagy was shown to be upregulated during DENV infection to promote viral production both in vitro and in vivo [6,7,8,9,10,18]. Autophagy inhibition has also been demonstrated by many studies to result in decreased DENV replication and progeny released from the host cells [7,8,10,19,20]. The mechanism involved in autophagy-mediated DENV production includes an enhanced lipid metabolism through lipophagy, resulting in increased ATP generation to promote efficient DENV replication [19]. Additionally, autophagy inhibition has been shown to affect DENV virion maturation by reducing the release of the pr peptide, resulting in decreased infectious DENV production [8]. Furthermore, DENV induces autophagy at an early stage of infection, but later on inhibits the autophagic flux to facilitate the production of DENV infectious particles [9]. In addition, DENV induces autophagy to augment the production of DENV-associated secretory autophagosomes that carry infectious DENV RNA and proteins from the host cells to the recipient cells, thereby evading neutralizing antibodies to initiate a new infection cycle in nearby cells [21]. Hence, the inhibition of DENV-induced autophagy is seen as critical to inhibit DENV infection. This study showed that Ka-003 inhibited DENV-induced autophagy in the host cells (Figure 4). Nevertheless, the exact molecular target of Ka-003 inside the cells and the step of the virus life cycle that was affected by the Ka-003 treatment remain to be identified.

Previous studies showed that DENV induces autophagy via the ATM, ER stress and AMPK pathways [36]. DENV induces the activation of ATM, a protein kinase that regulates DNA damage and oxidative stress response, leading to ER stress pathway activation via PERK, and resulting in ROS generation to promote autophagy in the host cells [37]. In response to the elevated ROS, it was also shown that ATM can suppress mTORC1, a nutrient/energy/redox sensor protein complex that inhibits autophagy in mammalian cells by activating AMPK, leading to autophagy induction [38]. Furthermore, activated AMPK was shown to be required for DENV-induced lipophagy and the depletion of AMPK blocked efficient DENV replication [39]. Whether Ka-003 inhibits DENV-induced autophagy by targeting factor(s) functioning in the aforementioned pathways is unknown and warrants further investigation.

As mentioned above, Ka-003 is a derivative of methyl cyclohexene that resembles those found in mulberry plants [31,32,33,34,35]. These include kuwanon G, kuwanon H, kuwanon X and mulbenofuran C. Kuwanon G treatment was previously shown to decrease ROS levels in LPS-treated Caco-2 cells, resulting in the reduced production of proinflammatory cytokines, including IL-1β and TNF-α, and increased cell survival [31]. Another study also demonstrated the antioxidative stress and anti-inflammatory activities of kuwanon G in HT22 cells induced to undergo injury through treatment with glycation end products [32]. A further characterization indicated that kuwanon G inhibited NF-κB activation in these cells, although the molecular target of kuwanon G remains unclear [32]. Interestingly, kuwanon G was also shown to possess antiviral activity against HCoV-229E in L-132 cells (IC_50_ = 5.61 μg/mL) [33], though the molecular mechanism remains uncharacterized. Importantly, kuwanon G and another methyl cyclohexene derivative isolated from mulberry plants, kuwanon H, were also shown to possess radical scavenging activity when tested using ABTS, superoxide and FRAP assays, which confirmed that these compounds could directly scavenge ROS [34]. In addition, kuwanon X and mulberrofuran C, both of which are also methyl cyclohexene derivatives found in mulberry plants, were shown to restrict HSV (IC_50_ = 1.5–2.5 μg/mL) by inhibiting NF-κB activation, although the exact mechanism remains unknown [35]. As Ka-003 shares a core structure with the aforementioned molecules and this study showed that Ka-003 inhibited autophagosome formation, and autophagosome formation requires ROS [40], whether Ka-003 can directly scavenge ROS resulting in autophagy inhibition is of interest and a subject of further investigation. In addition, whether the aforementioned methyl cyclohexene derivatives from mulberry plants also possess autophagy inhibitory and anti-DENV activities remains a topic of interest to be studied. It is critical to gain a better understanding of the underlying molecular mechanism elicited by Ka-003 and identify its cellular targets to aid in the improvement of Ka-003 efficacy through structural modifications.

## Data Availability

The datasets generated during and/or analyzed during the current study are available from the corresponding author on reasonable request.

## Supplementary Materials

The following supporting information can be downloaded at: https://www.mdpi.com/article/10.3390/v15102012/s1, Figure S1: generation and functional validation of GFP-LC3 knocked-in HeLa (GFP-LC3 KI HeLa) cells, Figure S2: generation and functional validation of GFP-LC3 knocked-in THP-1 (GFP-LC3 KI THP-1) cells, Figure S3: Ka-003 possesses low toxicity towards normal human kidney HK-2 cells, Figure S4: Ka-003 possesses low toxicity towards GFP-LC3 KI HeLa and THP-1 cells.
