# Supplementary material for: Novel Potent Autophagy Inhibitor Ka-003 Inhibits Dengue Virus Replication
Source: Viruses. 2023 Sep 27;15(10):2012. doi: 10.3390/v15102012 (PMC10611120; doi:10.3390/v15102012)
Supplement: Supplementary file 1 [file viruses-15-02012-s001.zip › viruses-2545422-supplementary.pdf]

A novel potent autophagy inhibitor Ka-003 inhibits dengue virus replication

Supplementary Methods

Cell toxicity assay

Human kidney normal cell HK-2 cells were obtained from ATCC. Cells were maintained in DMEM (Dulbecco's Modified Eagle Medium) supplemented with 10% FBS, 1% penicillin/streptomycin, and 1% HEPES at 37°C in 5% CO_2. At 24 h before treatment, HK-2 cells (1×10^4 cells) were plated onto 96-well plates. Cells were then treated with DMSO (negative control) or different concentrations of Ka-003 for 48 h. Cell toxicity was then measured by using the MTT assay (Sigma-Aldrich) per manufacturer's recommendation. For GFP-LC3 KI HeLa and THP-1 cells generated in this study, cells were plated and treated with Ka-003 as described above. Cell toxicity was then measured by using the MTS assay (Promega) per manufacturer's recommendation.

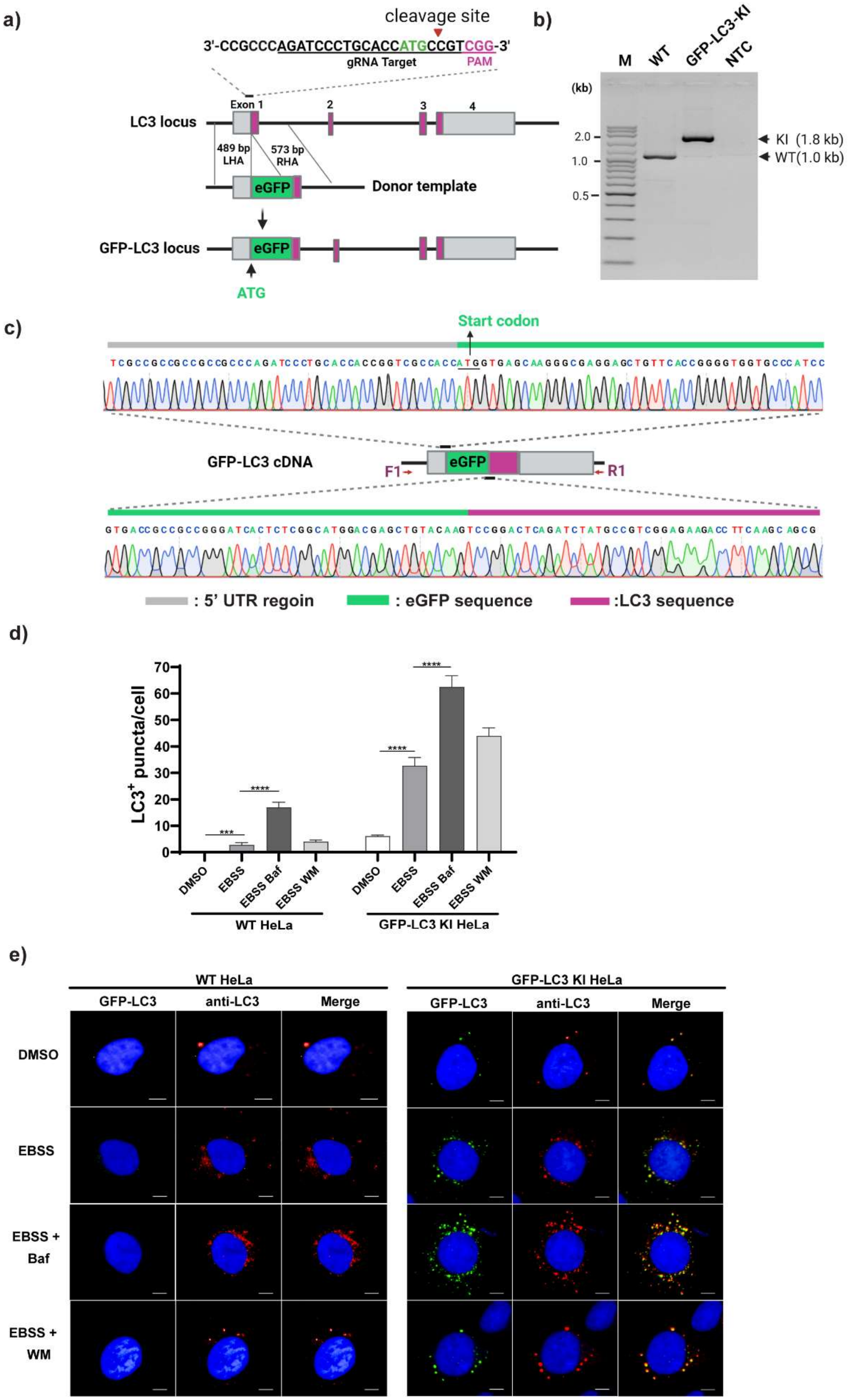

a)
cleavage site
3'-CCGCCCAGATCCCTGCACCATGCCGTCGG-3'
gRNA Target
PAM
Exon 1
2
3
4
LC3 locus
489 bp LHA
573 bp RHA
eGFP
Donor template
GFP-LC3 locus
ATG
b)
M
WT
GFP-LC3-KI
NTC
(kb)
2.0
1.0
0.5
KI (1.8 kb)
WT(1.0 kb)
c)
Start codon
GFP-LC3 cDNA
F1
R1
: 5' UTR regoin
: eGFP sequence
:LC3 sequence
d)
LC3+ puncta/cell
70
60
50
40
30
20
10
0
***
****
DMSO
EBSS
EBSS Baf
EBSS WM
WT HeLa
GFP-LC3 KI HeLa
e)
WT HeLa
GFP-LC3 KI HeLa
GFP-LC3
anti-LC3
Merge
DMSO
EBSS
EBSS + Baf
EBSS + WM

Figure S1. Generation and functional validation of GFP-LC3 KI HeLa cell line. (**a**) Schematic representation of CRISPR/Cas9-mediated knocked-in (KI) of eGFP at the N-terminus of the LC3 gene. The coding and non-coding regions of LC3 exons are represented by magenta and gray bars, respectively. The location of the specific single guide RNA targeting the start codon (ATG) is indicated by underlined letters. The predicted cleavage target site of Cas9 is shown with a red arrowhead. A donor template plasmid containing the eGFP gene flanked by a 489 bp left homology arm (LHA) and a 573 bp right homology arm (RHA) was used to induce homology-directed repair following CRISPR/Cas9-mediated double strand breaks, resulting in the integration of eGFP at the LC3 locus. (**b**) RT-PCR analysis of the integration of GFP in the indicated cells. Expected PCR product size: WT = 1.0 kb; KI = 1.8 kb. (**c**) cDNA sequencing analysis confirmed the successful generation of the GFP-LC3 KI HeLa cell line. A representative Sanger sequencing chromatogram of the cDNA around the 5' (upper panel) and 3' (lower panel) ends of the incorporated GFP is shown. The gray, green, and magenta bars represent the 5' UTR sequence, eGFP sequence, and LC3 gene, respectively. (**d-e**) The functionality of autophagy in GFP-LC3 KI HeLa cells compared to that of the wild type (WT) HeLa cells was assessed. WT and GFP-LC3 KI HeLa cells were subjected to different treatments: DMSO (negative control), EBSS (autophagy induction control), EBSS + wortmannin (WM; standard autophagy inhibitor control), or EBSS + bafilomycin A1 (Baf; standard autophagy flux inhibitor control) for 2 h. Following treatment, cells were fixed and stained with rabbit anti-LC3 antibody followed by goat anti-rabbit Alexa568-conjugated antibody per manufacturer's instruction. Cells were then stained with Hoechst 33342 and subjected to high-content image analysis. (**d**) Representative pictures of GFP-$LC3^+$ and $LC3^+$ puncta in the indicated cells with different treatments. (**e**)The number of $LC3^+$ puncta per cell was then analyzed. Data are the means $\pm$ SEM from three independent experiments; ***$p < 0.001$ and ****$p < 0.0001$, all relative to the respective control were determined by one-way ANOVA with Bonferroni's multiple comparison test. Bar 5 μm.

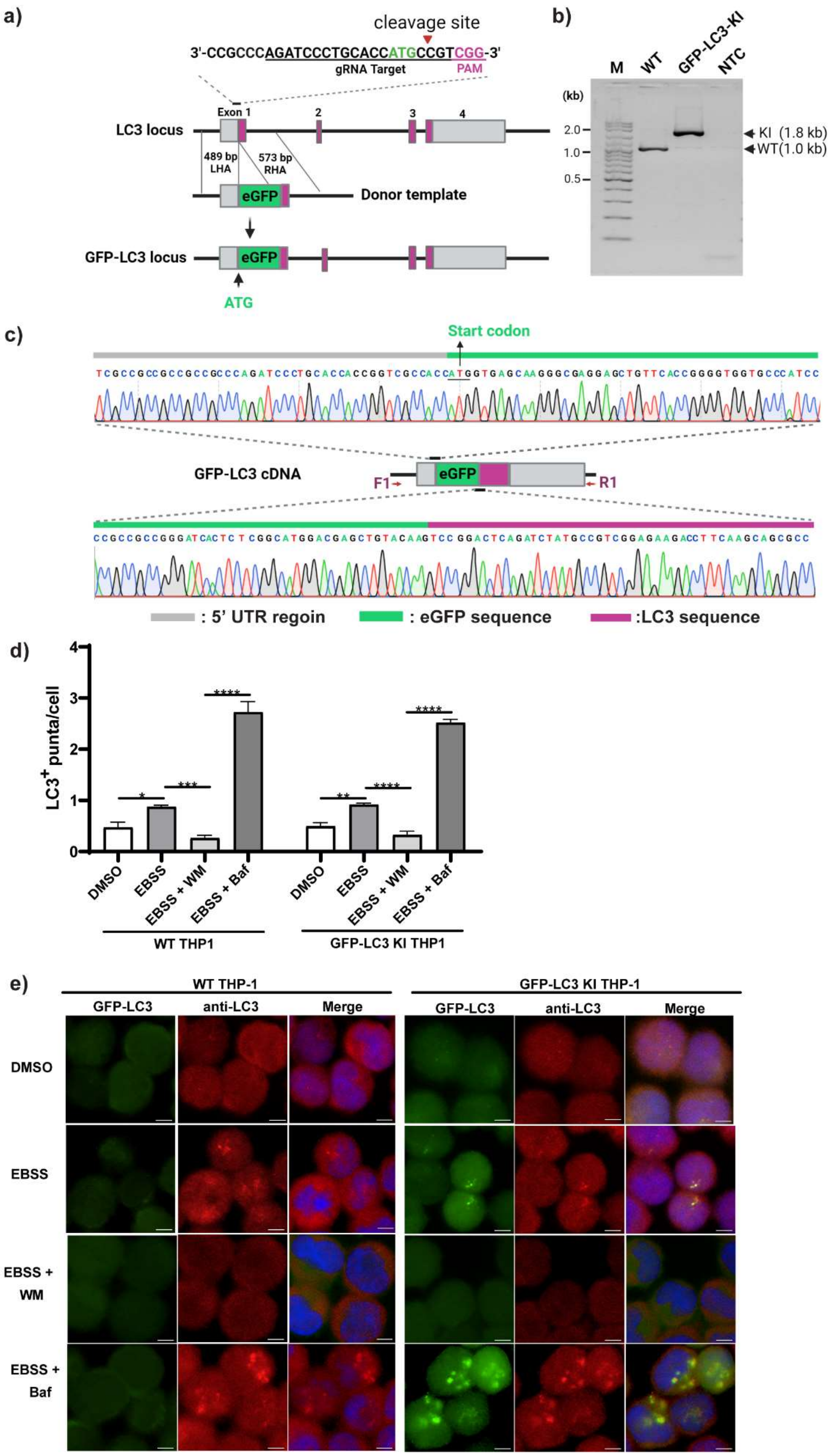
a)
cleavage site
3'-CCGCCCAGATCCCTGCACCATGCCGTCGG-3'
gRNA Target
PAM
Exon 1
2
3
4
LC3 locus
489 bp LHA
573 bp RHA
eGFP
Donor template
GFP-LC3 locus
ATG
b)
M
WT
GFP-LC3-KI
NTC
(kb)
2.0
1.0
0.5
KI (1.8 kb)
WT(1.0 kb)
c)
Start codon
GFP-LC3 cDNA
F1
R1
: 5' UTR regoin
: eGFP sequence
:LC3 sequence
d)
LC3+ punta/cell
0
1
2
3
4
*
***
****
**
****
****
DMSO
EBSS
EBSS + WM
EBSS + Baf
WT THP1
GFP-LC3 KI THP1
e)
WT THP-1
GFP-LC3 KI THP-1
GFP-LC3
anti-LC3
Merge
DMSO
EBSS
EBSS + WM
EBSS + Baf

Figure S2. Generation and functional validation of GFP-LC3 KI THP-1 cell line. (**a**) Schematic representation of CRISPR/Cas9-mediated knocked-in (KI) of eGFP at the N-terminus of the LC3 gene. Magenta and gray bars represent the coding and non-coding regions of LC3 exons, respectively. The underlined letters indicate the specific single guide RNA targeting the start codon (ATG). The red arrowhead indicates the predicted cleavage target site of Cas9. A donor template plasmid containing the eGFP gene, flanked by a 489 bp left homology arm (LHA) and a 573 bp right homology arm (RHA), was used to induce homology-directed repair after CRISPR/Cas9-mediated double-strand breaks, resulting in the integration of eGFP at the LC3 locus. (**b**) RT-PCR analysis of the GFP-LC3 KI in the indicated cells. Expected PCR product size: WT = 1.0 kb; KI = 1.8 kb. (**c**) cDNA sequencing analysis confirmed the successful generation of the GFP-LC3 KI THP-1 cell line. A representative Sanger sequencing chromatogram of the cDNA around the 5' (upper panel) and 3' (lower panel) ends of incorporated GFP is shown. The gray, green, and magenta bars represent the 5' UTR sequence, eGFP sequence, and LC3 gene, respectively. (**d-e**) The functionality of autophagy was assessed in the GFP-LC3 KI THP-1 cells compared to that of the wild type (WT) THP-1 cells. Cells were subjected to different treatments: DMSO (negative control), EBSS (autophagy induction control), EBSS + wortmannin (WM; standard autophagy inhibitor control), or EBSS + bafilomycin A1 (Baf; standard autophagy flux inhibitor control) for 2 h. Following treatments, cells were fixed and subjected to high-content image analysis as in Figure S1. (**d**) Representative images of GFP-LC3$^+$ and LC3$^+$ puncta in the eGFP-LC3 KI and WT THP-1 cells with the indicated treatments. (**e**) The number of LC3+ puncta per cell was then analyzed. Data represent the means ± SEM from three independent experiments; *$p < 0.05$, **$p < 0.01$, ***$p < 0.001$, ****$p < 0.0001$, all relative to the respective control, as determined by one-way ANOVA with Bonferroni's multiple comparison test. Scale bar: 5 µm.

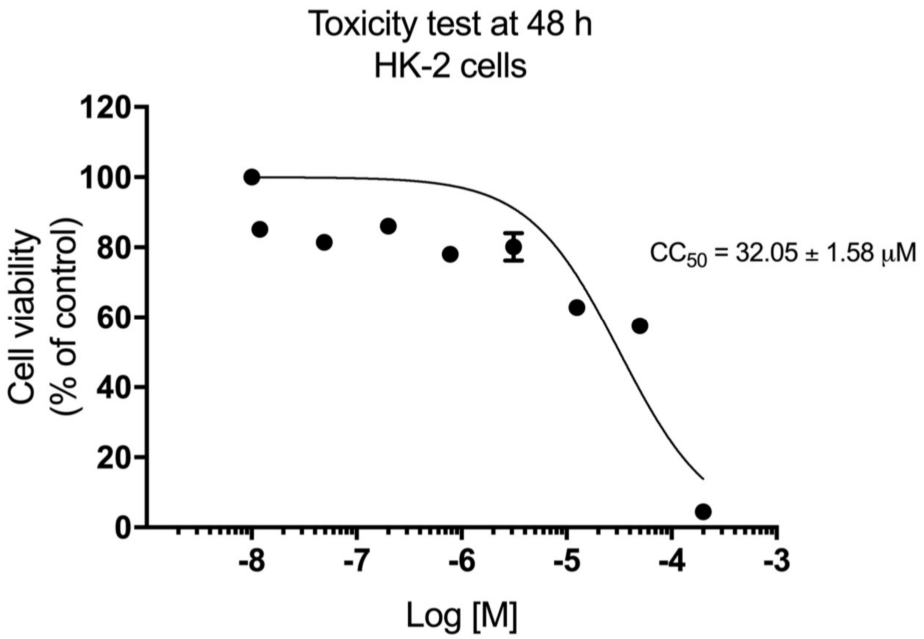

Figure S3. Ka-003 possesses low toxicity towards human kidney normal HK-2 cells. HK-2 cells were treated with DMSO (negative control) or varied concentrations of Ka-003 for 48 h and cell toxicity was measured by the MTT assay. Data are means ± SEM done in triplicate; the results were expressed relative to the DMSO control, defined as 100%.

a)

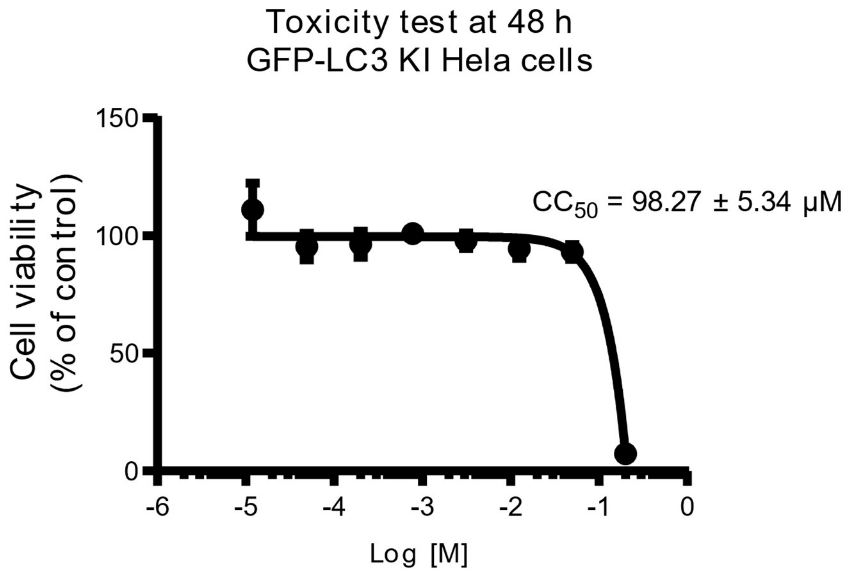

b)

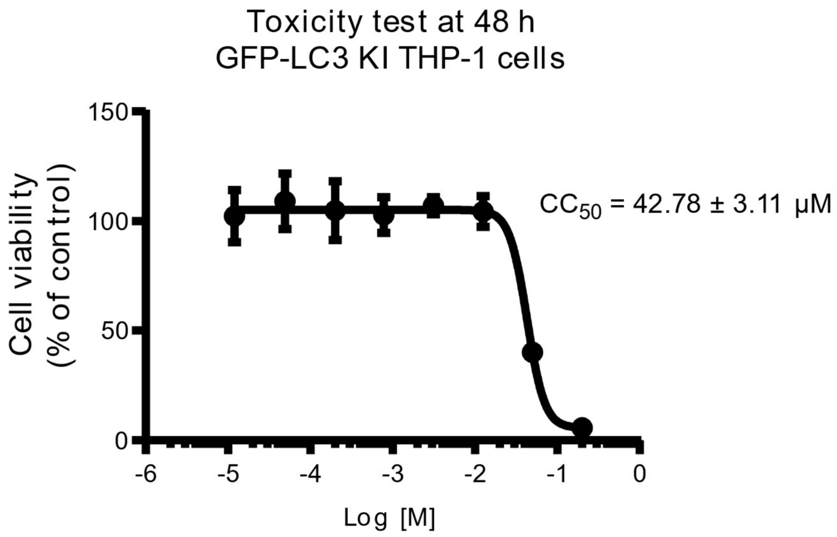

Figure S4. Ka-003 possesses low toxicity towards GFP-LC3 KI HeLa and THP-1 cells. (**a-b**) GFP-LC3 KI HeLa and THP-1 cells were treated with DMSO (negative control) or varied concentrations of Ka-003 for 48 h and cell toxicity was measured by the MTS assay. Data are means ± SEM done in triplicate; the results were expressed relative to the DMSO control, defined as 100%.
